# Dobutamine-Induced Myoclonus in a Patient With Acute Decompensated Heart Failure and Acute Kidney Injury: A Case Report and Literature Review

**DOI:** 10.7759/cureus.78295

**Published:** 2025-01-31

**Authors:** Saeed K Alzghari, Markus D Moore, Jacob C Mitchell

**Affiliations:** 1 Pharmacy, Texas Health Harris Methodist Hospital Fort Worth, Fort Worth, USA

**Keywords:** acute decompensated heart failure, acute kidney injury, adverse drug reaction, dobutamine, drug-induced myoclonus

## Abstract

Dobutamine is a potent β1 and a weak β2 adrenergic agonist used in the treatment of patients with acute decompensated heart failure or cardiogenic shock. Its positive inotropic effects enhance myocardial contractibility, leading to increased cardiac output. Myoclonus in patients receiving dobutamine is a rare adverse event that is not completely understood but seems to occur more commonly in patients with renal insufficiency. We present the unique case of a 61-year-old female receiving treatment for acute decompensated heart failure with acute kidney injury (AKI) who developed cortical myoclonus after 26 hours of a dobutamine infusion. Resolution occurred 36 hours after discontinuation of the dobutamine infusion. Valproic acid and diazepam were given to relieve the myoclonus. Clinicians should be aware that dobutamine-associated myoclonus is possible in patients with AKI.

## Introduction

Dobutamine is a synthetic catecholamine used in patients with acute decompensated heart failure, cardiogenic shock, or otherwise requiring inotropic support [1]. It works mainly by stimulating β1 receptors in cardiac myocytes, resulting in increased inotropy and chronotropy. Furthermore, dobutamine also has some activity on β2 receptors in vascular smooth muscle, leading to vasodilation. Lastly, dobutamine, to a lesser extent, has activity on α1 receptors in vascular smooth muscle leading to vasoconstriction; however, dobutamine's β2 activity negates its α1 activity. Overall, the combination of these effects by dobutamine leads to increased inotropy, chronotropy, and peripheral vasodilation that increases cardiac output to alleviate symptoms associated with heart failure [1]. The main adverse effects of concern with dobutamine include ventricular arrhythmias, tachycardia, and hypertension at typical doses. At higher doses, hypotension with dobutamine can occur due to increased vasodilation. It is hepatically metabolized and excreted via urine [1]. Myoclonus refers to a sudden involuntary twitching or spasm of a muscle. Common drugs associated with myoclonus include antipsychotics, anticonvulsants, opiates, and antibiotics [2]. A few case reports of dobutamine-associated myoclonus have been reported, with the majority of cases describing generalized myoclonus in patients with chronic kidney disease (CKD) [3-8]. A proposed mechanism of this phenomenon is theorized to be the stimulation of β2 receptors on skeletal muscle by dobutamine and accumulation in renal insufficiency [9]. Herein, we report on a case of dobutamine-induced myoclonus (DIM) in a patient with acute decompensated heart failure and acute kidney injury (AKI).

## Case presentation

A 61-year-old female with a significant past medical history of hypertension, gastroesophageal reflux disease, depression, gout, and chronic lung disease (2 liters of oxygen at baseline) was admitted for a right heart catheterization that confirmed acute on chronic decompensated heart failure with preserved ejection fraction (HFpEF). Her baseline laboratory findings were within normal limits, including calcium, magnesium, glucose, blood urea nitrogen (BUN), creatinine, and estimated glomerular filtration rate (eGFR), indicating normal renal function and electrolyte balance at day 1 admission (Table 1). The patient’s medication regimen upon admission was as follows (routes are considered oral unless otherwise noted): allopurinol 100 mg daily, carvedilol 6.25 mg twice daily, empagliflozin 10 mg daily, enoxaparin 40 mg subcutaneous twice daily, furosemide 80 mg intravenous (IV) every eight hours, hydralazine 10 mg IV every four hours as needed, hydrocodone-acetaminophen 7.5-325 mg every six hours as needed, losartan 100 mg daily, metolazone 2.5 mg daily, morphine 2 mg IV every four hours as needed, nitroglycerin 0.4 mg sublingual every five minutes as needed, ondansetron 4 mg IV every six hours as needed, and spironolactone 25 mg daily.

**Table 1:** Selected renal laboratory values throughout the duration of the hospital encounter *based on the Chronic Kidney Disease Epidemiology Collaboration (CKD-EPI) 2021 equation which does not use a race coefficient NR: not reported

Lab test (reference values)	Day 1	Day 2	Day 3	Day 4	Day 5	Day 6	Day 7	Day 8
Calcium (8.5-10.5 mg/dL)	9.6	NR	9.5	9.4	9.9	9.1	9.6	9.9
Magnesium (1.3-2.1 mEq/L)	1.5	1.7	1.7	1.8	2.4	2.3	2.2	1.7
Glucose (70-100 mg/dL)	83	NR	108	95	106	89	106	84
Blood urea nitrogen (7-26 mg/dL)	17	NR	37	63	70	69	61	60
Creatinine (0.6-1.3 mg/dL)	0.8	NR	2.0	3.5	3.6	2.7	1.8	1.5
Estimated glomerular filtration rate (≥60 mL/min/1.73m^2^)*	84	NR	28	14	14	19	32	39

On the third day of admission, the patient developed AKI with a rise in creatinine from 0.8 mg/dL at baseline to 2.0 mg/dL. Her BUN also increased to 37 mg/dL. Consequently, furosemide, metolazone, carvedilol, and spironolactone were discontinued. Subsequently, cardiology initiated IV dobutamine at 5 mcg/kg/min for inotropic support.

On the fourth day of admission, hypotensive episodes required albumin administration. In turn, the IV dobutamine dose was reduced to 2.5 mcg/kg/min. A continuous IV furosemide infusion was started at 5 mg/hr once the patient's blood pressure stabilized to manage the patient’s fluid overload. Nephrology was consulted to assess decreased renal function, as creatinine increased to 3.5 mg/dL, BUN increased to 63 mg/dL, and eGFR declined to 14 mL/min/1.73 m².

The following day, the patient began experiencing severe twitching and muscle spasms, primarily affecting the face with a more pronounced effect on the right side. Initial attempts to manage these symptoms with two doses of oral methocarbamol 750 mg were unsuccessful. Diazepam 5 mg IV every six hours as needed was also administered but only provided temporary relief. The spasms were suspected to be related to either electrolyte imbalance due to furosemide or a side effect of dobutamine. Furosemide and dobutamine were held, and calcium gluconate IV 2 g once and magnesium sulfate IV 2 g once were administered to address potential metabolic derangements. The myoclonus developed approximately 26 hours into the dobutamine infusion, and the infusion was given for a total of 30 hours.

Neurology was consulted and confirmed whole facial twitching, right more than left, with some twitching of her extremities noted. A computed tomography scan of the brain was unremarkable. An electroencephalogram showed no epileptiform activity, and left facial twitching was present throughout the study associated with muscle artifacts consistent with myoclonic phenomenon. Given the refractory nature of the twitching, a loading dose of valproic acid 2,500 mg IV once (20 mg/kg) was administered in addition to the as-needed IV diazepam. Nephrology and cardiology teams suspected DIM, supported by the absence of significant magnesium and calcium abnormalities. Magnesium levels had stabilized with treatment (2.4 mg/dL), and calcium was within normal limits (9.9 mg/dL).

By the sixth day, approximately 36 hours after dobutamine infusion discontinuation, the patient’s myoclonic episodes had resolved. The patient's renal function showed slight improvement, with a creatinine level of 2.7 mg/dL and eGFR of 19 mL/min/1.73 m². The patient’s BUN remained elevated at 69 mg/dL. Neurology concluded that the myoclonus was not related to seizures. However, the patient reported feeling extremely fatigued, likely secondary to the cumulative effects of diazepam administered during the myoclonic episodes.

Laboratory results showed improving renal function the following day, with a creatinine level of 1.8 mg/dL, BUN of 61 mg/dL, and eGFR of 32 mL/min/1.73 m². This improvement allowed the resumption of oral furosemide 40 mg daily to manage the patient’s ongoing fluid overload. The patient's clinical condition had improved, with no recurrence of myoclonus or twitching.

The patient's renal function continued to recover prior to discharge on day 8, with creatinine down to 1.5 mg/dL, BUN to 60 mg/dL, and eGFR improving to 39 mL/min/1.73 m². Electrolyte levels, including calcium and magnesium, were monitored closely and remained within normal limits. The clinical picture showed overall improvement regarding the patient’s heart failure, and the patient was discharged with a heart failure treatment plan to be managed on an outpatient basis.

## Discussion

We present a unique case of DIM in a patient with acute decompensated heart failure with AKI. The Naranjo Scale, a questionnaire used to determine if an adverse event was caused by a drug or another factor, produced a score of 7, deeming the reaction probable [10]. When reviewing the literature, we searched PubMed and Google Scholar for articles published through January 2025 using the keywords "dobutamine-induced myoclonus" and "dobutamine myoclonus." We identified six reports that included 11 patients experiencing DIM as summarized in Table 2 [3-8]. The subjects’ ages ranged from 51 to 74 years, and 6 out of the 11 subjects were male. All patients had either CKD or end-stage renal disease (ESRD). Cardiovascular co-morbidities included seven patients with congestive heart failure, three patients with ischemic cardiomyopathy (CM), and one patient with non-ischemic CM. Dobutamine dosing ranged from 2.5 µg/kg/min to 20 µg/kg/min. The onset of DIM occurred as early as 12 hours or as late as three weeks; however, the majority of cases reported an onset between 12 to 72 hours, and our patient experienced DIM onset at 26 hours. There was no correlation between the severity of renal dysfunction and the onset of DIM. Symptom resolution ranged from 30 minutes to 3 days once the dobutamine infusion was stopped. Our patient similarly had HFpEF, and the onset as well as the duration of DIM mirrors previous reports that included heart failure patients [6,8]. Furthermore, anti-seizure medications, including clonazepam and levetiracetam, were also utilized to alleviate symptoms [6,7]. However, the selection of valproic acid in our particular case was utilized because it is believed to treat cortical myoclonus through augmentation of the inhibitory influence of gamma-aminobutyric acid (GABA) [11]. It is also thought to somewhat have the ability to decrease the excitation of glutamate as well as alter ionic conductance. Diazepam was used in our case as an adjunctive treatment to valproic acid and is thought to exert its effect by enhancement of GABAergic neurotransmission by benzodiazepine receptors [11].

**Table 2:** Reports of dobutamine-induced myoclonus AF: atrial fibrillation; BPH: benign prostatic hyperplasia; CHF: congestive heart failure; CKD: chronic kidney disease; CM: cardiomyopathy; COPD: chronic obstructive pulmonary disease; CrCl: creatinine clearance; ESRD: end-stage renal disease; F: female; GERD: gastroesophageal reflux disease; HF: heart failure; HT: hypothyroidism; HTN: hypertension; ICD: implantable cardioverter-defibrillator; M: male; NR: not reported; PD: peritoneal dialysis; P-gp: P-glycoprotein; PH: pulmonary hypertension; T2DM: type 2 diabetes mellitus

Report	Sex(es), age(s)	Comorbidities	Dobutamine dosing (µg/kg/min)	Onset and duration of myoclonus	Treatment for symptoms of myoclonus	Potential Inhibitors of P-gp on patient’s profile
Sunnaa et al., 2024 [3]	F, 73	CKD, ischemic CM	2.5	Appeared 24 hours after starting dobutamine infusion, improved 12 hours after discontinuation, and completely disappeared after 24 hours	NR	NR
Lee et al., 2024 [4]	F, 51	Non-ischemic CM, CKD, biventricular ICD, HT	3	Appeared 12 hours after starting dobutamine infusion and abated within 30 minutes of discontinuation	NR	Atorvastatin, sertraline
Noel et al., 2022 [5]	F, 64	HT, pulmonary sarcoidosis, sleep apnea, secondary PH, paroxysmal AF, dilated non-ischemic CM, ESRD on PD	5	Appeared after three weeks on home dobutamine infusion and abated two days after discontinuation	NR	Atorvastatin, cinacalcet
Zamora and Shpiner 2021 [6]	M, 71	CKD, T2DM, depression, AF, HTN, systolic HF	5	Appeared within 24 hours of starting dobutamine infusion and abated once infusion was stopped	Intravenous levetiracetam showed moderate improvement	Sertraline
Boord and Benson, 2007 [7]	M, 65	Ischemic CM, ESRD on PD, AF, carotid artery disease, COPD, HTN, T2DM, chronic anemia, gout, GERD, BPH	3	First instance: appeared on the third day after starting dobutamine infusion and abated 2-3 days after discontinuation; second instance: appeared on the second day after starting dobutamine infusion and abated three days after discontinuation	Oral clonazepam partially relieved symptoms	Amiodarone, carvedilol, simvastatin
Wierre et al., 2004 [8]	Four M and two F, 68.6 ± 6.35	CHF, severe renal insufficiency (CrCl 15.5 ± 2.5 mL/min/1.73m^2^)	10-20	Appeared on the second or third day after starting dobutamine infusion, persisted during infusion, and abated two days after discontinuation	NR	NR

Regarding the reason for DIM, Wierre et al. proposed two mechanisms that included reduced metabolism of dobutamine catechol-O-methyltransferase (COMT) and an increase in blood-brain barrier (BBB) permeability caused by p-glycoprotein (P-gp) inhibition at the BBB leading to increased concentration in the central nervous system [8]. The inhibition of P-gp at the BBB is theorized to increase the concentration of dobutamine in the central nervous system and, in turn, lead to stimulation of β2 receptors on skeletal muscle, causing myoclonus [3,8]. Metabolism of dobutamine occurs through O-methylation by COMT. In uremic patients, O-methylation could be altered by non-competitive red blood cell COMT inhibition due to greater concentrations of endogenous methyl acceptors in the plasma of uremic patients than in non-uremic patients [12]. However, myoclonus in uremia is theorized to occur by the excitatory effect of N‐methyl‐d‐aspartate receptors and an inhibitory effect on GABA receptors that can lead to increased neuronal excitability [13]. Our patient did experience significant elevations in BUN while in AKI, but the patient's BUN remained elevated throughout her hospital stay suggesting that the discontinuation of dobutamine led to the resolution of myoclonus. Although our patient was on carvedilol, a potential P-gp inhibitor and a potential risk factor for DIM, it was discontinued prior to the initiation of dobutamine. Future studies of DIM could include potentially starting a dobutamine infusion at a lower rate in patients with renal insufficiency.

## Conclusions

Clinicians must be aware of the potential side effects of myoclonus in heart failure patients with AKI, CKD, or ESRD on dobutamine receiving inotropic support when managing similar patients in the future. Clinicians should closely monitor renal function, electrolytes, and signs and symptoms of myoclonus. Adjusting the dobutamine rate may help alleviate symptoms of myoclonus. Valproic acid and diazepam may also be useful in ameliorating symptoms associated with DIM. This case adds to the growing body of evidence that DIM can occur in AKI patients. Given that significant myoclonus developed in this case, collaboration among cardiologists for adjusting inotropes, neurologists for symptom control, and nephrologists for renal optimization is crucial in the evaluation and management of DIM.

## References

[1] Ashkar H, Adnan G, Patel P, Makaryus A (2025). Dobutamine. StatPearls [Internet].

[2] Janssen S, Bloem BR, van de Warrenburg BP (2017). The clinical heterogeneity of drug-induced myoclonus: an illustrated review. J Neurol.

[3] Sunnaa M, Kerolos M, Satterfield M, Shakoor R, Koirala S, Snell J (2024). Dobutamine-induced myoclonus: acute myoclonus with continuous dobutamine and chronic kidney disease. AIM Clinical Cases.

[4] Lee AY, Barforoshi S, Singh A, Shrestha R, Ha J, Kittleson M (2024). Dobutamine-induced myoclonus in a patient with advanced heart failure and chronic kidney disease. JACC Case Rep.

[5] Noel E, Fayoda B, Rabbani R, Benjamin YS, Lee J, Gillespie A (2023). Dobutamine-induced myoclonus in a peritoneal dialysis patient: case report. Kidney Med.

[6] Zamora M, Shpiner D (2021). Drug-induced myoclonus in a patient on continuous dobutamine infusion (5125). Neurology.

[7] Boord A, Benson B (2007). Myoclonus associated with continuous dobutamine infusion in a patient with end-stage renal disease. Am J Health Syst Pharm.

[8] Wierre L, Decaudin B, Barsumau J, Vairon MX, Horrent S, Odou P, Azar R (2004). Dobutamine-induced myoclonia in severe renal failure. Nephrol Dial Transplant.

[9] Ahrens RC (1990). Skeletal muscle tremor and the influence of adrenergic drugs. J Asthma.

[10] Naranjo CA, Busto U, Sellers EM (1981). A method for estimating the probability of adverse drug reactions. Clin Pharmacol Ther.

[11] Caviness JN (2014). Treatment of myoclonus. Neurotherapeutics.

[12] Pazmiño PA, Weinshilboum RM (1980). Methyl conjugation in uraemia: catechol-O-methyltransferase. Br J Clin Pharmacol.

[13] Rani P, Sharma R, Tater P (2021). Reversible facial myoclonus in uremia. Mov Disord Clin Pract.

